# Evaluation of Acellular Dermal Matrix (ADM) as a Scaffold for Adipose-Derived Stem Cell Transfer in the Rat Model

**DOI:** 10.29252/wjps.10.2.67

**Published:** 2021-05

**Authors:** Maryam Jahanian, Sara Hoseini, Amir Atashi, Mohsen Saberi, Seyyed Aboozar Hoseini, Kambiz Mozaffari, Mohammad Javad Fatemi

**Affiliations:** 1 Burn Research Center, Hazrat Fatima Hospital, Rehabilitation Research Center, School of Rehabilitation Sciences, Iran University of Medical Sciences, Tehran, Iran.; 2 Burn Research Center, Rajaie Cardiovascular Medical and Research Center, Iran University of Medical Sciences, Tehran, Iran.; 3 Stem Cell and Tissue Engineering Research Center, Shahroud University of Medical Sciences, Shahroud, Iran.; 4 Community Medicine, Quran and Hadis Research Center, Faculty of Medicine, Baqiyatallah University of Medical Sciences Tehran, Iran.; 5 Burn Research Center, Hazrat Fatima Hospital, Iran University of Medical Sciences, Tehran, Iran.; 6 Rajaie Cardiovascular Medical and Research Center, Iran University of Medical Sciences, Tehran, Iran.; 7 Department of Plastic & Reconstructive Surgery, Burn Research Center, Hazrat Fatima Hospital Iran University of Medical Sciences, Tehran, Iran.

**Keywords:** Stem cell, Tissue engineering, Acellular Dermis

## Abstract

**BACKGROUND:**

This study was designed for the evaluation of Acellular Dermal Matrix (ADM) as a scaffold for adipose-derived stem cell transferring in the rat model.

**METHODS:**

This experimental study was done in the Burn Research Center of Iran University of Medical Sciences and Bonyakhteh Research Center, Tehran, Iran according to the standards of laboratory animals. Overall, 26 healthy Sprague-Dawley rats were used. Two of them were used to prepare ADM. In group one, the first wound on each, rat was spread with the mixture of fibrin gel and autologous stem cell. Only the stem cells combined with fibrinogen were spread on the other wound. In group two, the first wound on each rat was covered only with ADM, and the second wound was covered with gauze Vaseline. To perform sampling we used observation and photography at 7-30 days. Overall, 48 samples were taken of all the rats using skin punch biopsy on the 30th day for histopathology evaluation.

**RESULTS:**

There were significant differences in each group; however, the difference between different groups on days was not significant. In pathology, epithelialization, vascularization, the amount of collagen, collagen arrangement, the number of fibroblasts, and inflammation indices were investigated. The total score in each group was used for analysis. In statistical analysis, there was no pathology score difference among groups.

**CONCLUSION:**

**U**sing stem cells with or without ADM could not enhance the process of wound healing or improve pathology indices.

## INTRODUCTION

Numerous functional and physiological difficulties are caused by full-thickness skin wounds resulted from extensive burns, chronic ulcers, and acute trauma^1^^-^^3^. Plastic and reconstructive surgery always faces the challenge of wound healing enhancement. A complex process is involved in cutaneous wound healing. This process is a combination of molecular and biological events such as migration of cells, their proliferation as well as deposition and angiogenesis of extracellular matrix (ECM) and its remodeling^4^^-^^6^.

In recent years, there have been significant advancements in comprehending pathophysiological aspects of wound healing as well as developing novel therapeutic techniques; however, there exist challenges during the healing of chronic wounds. The most critical factors that contribute to unsatisfactory wound healing are a decrement in neovascularization and a decrease in cytokines released by local inflammatory cells^5^^, ^^7^^, ^^8^. 

A long-term self-renewal capability has been observed in adult stem cells. They are also able to be differentiated into different types of cells and release various growth factors for angiogenesis simulation^9^^, ^^10^. The potential of bone marrow mesenchymal cells (BMSCs) for wound healing acceleration has been reported by several researchers^11^^-^^13^. Although stem cells can be isolated from different tissues, adipose tissue is an interesting source. Typically, there are more stem cells in fat tissue than bone marrow and peripheral blood. Moreover, fat is easily accessible in large quantity and harvesting would be less complex and risky^14^^-^^16^.

Adipose-derived stem cells ADSCs show properties similar to BMSCs and are alternative to pluripotent cells^17^^-^^19^. Additionally, these cells offer numerous advantages over BMSCs, such as multipotency independent of serum source, ease of isolation, rapidity of expansion, and relative abundance^20^^, ^^21^. The beneficial effects of stem cells on the acceleration of wound healing have been investigated in some studies. Stem cells with paracrine secretion and differentiation abilities have been reported to promote the healing process^1^^, ^^22^. However, there are controversial results regarding their true effectiveness in clinical trials.

Intervention in the healing process by stem cells may be by local application, local injection into or around the wound, and systemic injection^23^^-^^25^. When the local application is the route, most of the time a scaffold or career is used for transferring stem cells. Different scaffolds were introduced, yet chitosan is the most common^26^^, ^^27^. As a result of several advantages including cell proliferation promotion and removing exudates from the wound site, scaffolds have been considered as a promising substrate for tissue engineering, especially for repairing damaged skin^28^^-^^33^.

In several studies, autologous split-thickness skin grafts were used to cover Integra, Matriderm, or acellular dermal matrix (ADM) by a one-step process^34^^-^^38^. To treat deep burns, Srivastava et al.^39^ used ADM as a dermal substitute, however, there are limitations in the availability of cadaver skin to produce ADM. The effect of using a xenogeneic dermal substitute based on porcine ADM on wound healing rates was evaluated.

Nie et al. ^40^ demonstrated accelerated wound closure of ASCs seeded ADM scaffolds by the enhancement of tissue regeneration as well as increasing epithelialization.

Chunlei et al. ^10^ suggested that adipose-derived stem cells (ADSCs) could act as promising alternatives for the improvement of the wound healing process by differentiation and angiogenesis. Moreover, using a scaffold seeded by the stem cells was shown to facilitate the introduction of the cells to the local ischemia environment providing a framework for supporting the regenerative capacity of the cells. The introduction of allogeneic ADSCs to an ADM scaffold could be suggested as a beneficial method for cutaneous wound treatment. ADSCs noticeably reduce the size of the wound and accelerate the process of re-epithelialization ^41^. 

There are limited studies regarding using stem cells with or without ADM; therefore, in this study, ADM as a scaffold for stem cell transport, support, and release in full-thickness wounds in animal models of rats was evaluated to minimize wound healing time and improve surgical site performance, especially in patients with high surgical range.

## MATERIALS AND METHODS

The study was confirmed by the Ethical Committee of Iran University of Medical Sciences and the standards of care and use of animals in research were followed. 

This experimental study was done in the Burn Research Center of Iran University of Medical Sciences and Bonyakhteh Research Center, Tehran, Iran. Twenty-six healthy Sprague-Dawley rats, weighing approximately 300 to 350 g were used. Two rats were used for ADM preparation, 12 rats were assigned for stem cell and scaffold group and 12 rats for a scaffold. All rats were anesthetized with an intramuscular injection of 10% ketamine 90 mg/kg (Alfasan Woerden, Netherland) and Xylazin 2%, 9 mg/kg (Alfasan Woerden, Netherland). All procedures were performed under sterile condition and all rats received antibiotic prophylaxis. 

The total back skin of two rats was used for ADM production in Transplant Tissue Research Center. Moreover, the inguinal fat of 12 rats in the first group was harvested and used for autologous fat-derived stem cells. All rats and their samples numbered precisely and the specimens were sent in normal saline to the cell research center for isolation and proliferation of stem cells. 

***Acellular dermis production***

The skin was free of subcutaneous fat and hair follicles during the Physical treatment. The tissue was treated for 24 h in a solution of sodium chloride with an antibiotic cocktail Penn. Then, epidermolysis and isolation of epidermal cells were carried out. Next was secondary physical treatment to cleanse the epidermal layer. Trypsin and Triton chemical treatments were then done, followed by rinsing products by deionized water. Finally, the products were kept in the freezer at -80 °C. Lyophilized samples were segmented into two types based on their thickness under the biological safety cabinet. Boxes containing brochures, product testers, and corresponding labels were transferred to the International Atomic Energy Agency. After delivery from the Atomic Energy Organization and microbiological test, the products were delivered to the quality control unit.

***Isolation and culture of adipose-derived mesenchymal stem cells***

Immediately after the transfer of adipose tissue to the lab, it should be as much homogenized as possible and washed three times by PBS buffer containing antibiotics (penicillin) ^42^.

Then the adipose tissue is treated with collagenase type 1 (Invitrogen) for 3 h at 37 degrees centigrade. Next, by adding FBS (Invitrogen), neutralization of the enzyme is performed and the cells are centrifuged at 1200 rpm for 10 minutes.

 Using a lubricating solution (Stem cell technologies), red blood cells in cell plates are lysed, and then centrifuged^43^. The cells in T25 flasks containing DMEM and 15% FBS, antibiotics, penicillin/streptomycin, and glutamine are grown. The medium was replaced three times per week and the third passage of cells was used to perform the sequence of steps.

***Protocol for fibrin gel*** ***preparation***

The material to prepare a fibrin gel was purchased from Evicell Company. After the third passage, to expose to the fibrin glue, the rat mesenchymal stem cells, were isolated by trypsin/EDTA (Invitrogen. pipette single cells and the cells were centrifuged at 1200 rpm. The cell plate was washed by PBS buffer and centrifuged again in the same round. A volume of 200 ml fibrinogen concentration of 40 mg/ml was added to the washed cell plates and mixed completely to the enzyme thrombin concentrations of recombinant 1000 IU/mL was added and vortexed immediately. The mixture was immediately transferred to an incubator at 37 °C and after 3 min fibrin gel containing rat mesenchymal stem cells was prepared. The fibrin gel is used for two purposes: First, the fibrin gel forms a three-dimensional nanofiber structure that is biologically safe. This holds the mesenchymal stem cells in Situ until total fibrin holds degradation of the gel occurs. Second, the fibrin degradation by-products induce angiogenesis in the wound causing faster wound healing.

***Second surgical procedure***

All rats were anesthetized and two separate 2x2 wounds were created in the back of each rat. In 12 rats, one of the wounds was spread with a mixture of fibrin gel and autologous stem cell, the cell number was 800 thousand cells in a volume of 50 ml. Then ADM was placed on the cell-gel mixture, and the other wound, only the stem cells were spread. To insert the stem cells into the wound it is required to combine the cells with fibrin gel. In group two, the first wound on each rat was covered with ADM but without stem cells and the second wound covered with gauze Vaseline. All wounds were evaluated by photography with 7 d interval till 28 d after surgery. The photos were taken by a digital NICON D 300 camera and 18-200mm lens, from a distance of 80cm. The wound area was measured in centimeters, by Image J, v1.40 (Wayne Rasband, NIH, USA) (NIH, USA) after calibration.

On day 30, a punch biopsy of all 48 wounds was performed. After finishing the procedure, the rats were euthanized by carbon dioxide according to the standard principles. The samples were placed in a 10% formalin and paraffin blocks were prepared after tissue processing. Four micron sections were made and the slides were stained using Hematoxylin & Eosin (H&E) staining method. A light microscopy study was done at x400 and x100 magnifications. Granulation tissue depth, the extent of epithelialization, fibroblastic proliferation, and neovascularization were evaluated in fields measuring 125 × 125 cm.

***Data analysis***

The information was analyzed using the SPSS survey (Chicago, IL, USA) and all the data was presented as MEAN +/- SD. For comparison, an average of 4 group analysis of variance ANOVA was used. Differences in the results were considered statistically significant when *P*<0.05.

## RESULTS

**Fig. 1:**
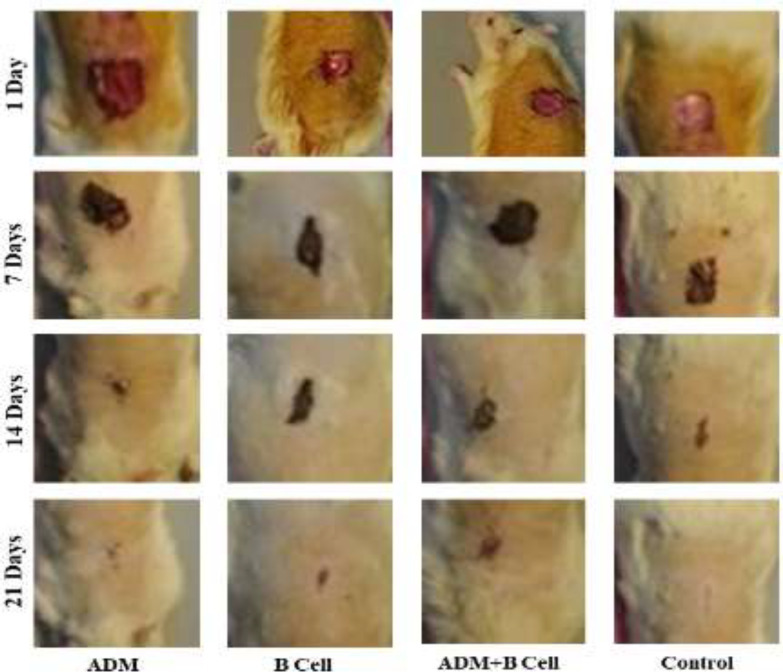
Wound healing progress in different groups after 1, 7, 14, and 21 days

Two rats of the control and ADM groups died, therefore, the data of 10 rats were analyzed and reported. Figure 1 shows the progress of the wound healing process in ADM, B cells, ADM+B cells, and control groups after 1, 7, 14, and 21 days. Wound healing proceeded in all groups over time, however, the wound healing process was faster in the group where ADM dressing was used. Hence, the wound size was smaller in the ADM group compared to other groups after 14 days. In the control group, where the wound was normally bandaged with Vaseline, good healing progress was observed after 14 and 21 days. The healing process was slower and the wound areas were larger in groups of B cells and ADM+B cells. 

**Fig. 2:**
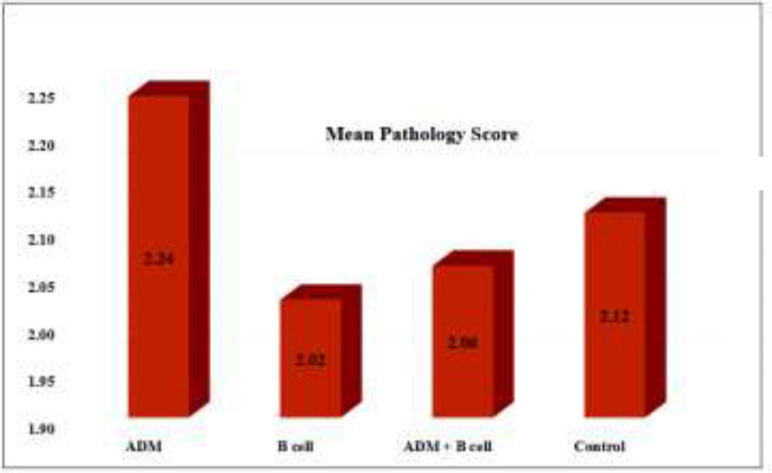
Mean pathology score for different groups

The average area of the wound surface with the standard deviations for all samples are shown in Table 1. According to the Repeated Measurement ANOVA test determined for 4 repetitions, the size of the wounds decreased during 21 d in each group. The obtained statistical results of wound size analysis are provided in Table 2. Accordingly, there was a statistically significant difference between wound sizes on different days in each group. Despite this, no statistically significant difference was observed between different groups for each day. Furthermore, pathological scoring was carried out based on epithelialization, vascularization, the amount and arrangement of collagen, the number of fibroblasts, and inflammation indices. Figure 2 shows the mean pathology score of the control, ADM, B cells, and ADM+B cells. A score of 1 to 4 was given to each group from lowest to highest. The scoring was vice versa (1 to 4 from highest to lowest) for the inflammation index due to its undesirable effect. There is a numerical difference between the groups, however, there were no significant differences in the pathology scores of the groups in the statistical analysis.

**Table 1:** The average area of wound surfaces for all samples

**Descriptive **		**N**	**Mean**	**Std.** **Deviation**	**Std. ** **Error**	**95% Confidence Interval for Mean**		**Min.**	**Max.**
						**Lower Bound**	**Upper Bound**		
**Initial area**	control	10	3.75120	1.439102	.455084	2.72173	4.78067	2.274	7.056
	ADM	10	3.67050	.797553	.252208	3.09996	4.24104	2.324	5.046
	Cell	12	3.39042	.624322	.180226	2.99374	3.78709	2.398	4.791
	ADM+Cell	12	4.30917	1.264314	.364976	3.50586	5.11247	2.916	6.840
	Total	44	3.78664	1.094837	.165053	3.45378	4.11950	2.274	7.056
**Area after 7 d**	control	10	2.16160	.653994	.206811	1.69376	2.62944	1.284	3.416
	ADM	10	2.34860	.574283	.181604	1.93778	2.75942	1.572	3.206
	Cell	12	1.86925	.275361	.079490	1.69429	2.04421	1.220	2.228
	ADM+Cell	12	2.01983	.490652	.141639	1.70809	2.33158	1.099	2.673
	Total	44	2.08570	.520796	.078513	1.92737	2.24404	1.099	3.416
**Area after 14 d **	control	10	.49210	.395348	.125020	.20928	.77492	.143	1.302
	ADM	10	.85090	.638926	.202046	.39384	1.30796	.189	2.274
	Cell	12	.69050	.359517	.103784	.46207	.91893	.304	1.340
	ADM+Cell	12	.61667	.301130	.086929	.42534	.80800	.168	1.053
	Total	44	.66173	.436154	.065753	.52912	.79433	.143	2.274
**Area after 21 d **	control	10	.08070	.058995	.018656	.03850	.12290	.004	.188
	ADM	10	.19250	.185191	.058563	.06002	.32498	.029	.538
	Cell	12	.17425	.122159	.035264	.09663	.25187	.081	.527
	ADM+Cell	12	.17442	.106922	.030866	.10648	.24235	.039	.370
	Total	44	.15718	.128309	.019343	.11817	.19619	.004	.538

**Table 2:** ANOVA result of different groups after 7, 14, and 21 days

**ANOVA**		**Sum of Squares**	**df**	**Mean Square**	**F**	**Sig.**
**Initial area**	Between Groups	5.308	3	1.769	1.531	.221
	Within Groups	46.235	40	1.156		
	Total	51.543	43			
**Area after 7 d**	Between Groups	1.363	3	.454	1.764	.169
	Within Groups	10.300	40	.257		
	Total	11.663	43			
**Area after 14 d **	Between Groups	.680	3	.227	1.209	.319
	Within Groups	7.500	40	.187		
	Total	8.180	43			
**Area after 21 d **	Between Groups	.078	3	.026	1.652	.193
	Within Groups	.630	40	.016		
	Total	.708	43			

## DISCUSSION

A promising strategy to repair skin wounds is stem cell tissue engineering. Stem cells possess various potentials and it is possible to drive these cells from different tissues. They have exhibited their therapeutic effects for different diseases including wounds, stroke, myocardial infarction, and bone fractures^44^^-^^47^. ADM, which is being used recently, does not have cells therefore it will not be rejected in the body and blood perfusion will be provided. This tissue can also be transplanted as an allograft and then covered with a thin layer of autologous skin. If this layer functions as a stem cell carrier wound healing will probably be faster^24^^, ^^48^^, ^^49^.

Stem cells can be transplanted into a defect area for the enhancement of wound healing through inflammation modulation, angiogenesis promotion, the proliferation of host cells, improvement of granulation formation, and re-epithelization^50^^-^^52^. However, some results do not certify this hypothesis^53^. 

MSCs seeded ADM has good potential for full-thickness skin wound treatment through the improvement of ECM remodeling, neovascularization, and complete skin regeneration^1^. Huang et al.^54^ studied the effect of ADSCs seeded ADM on full-thickness defect healing in a murine model. The prepared scaffold enhanced wound healing process, promoted angiogenesis, and increased the re-epithelialization rate in comparison with ADM. The scaffolds’ vascularization capacities could be promoted using ADSCs^55^. The scaffold's vascularization capacities and their effect on the angiogenic potential of ADSCs can influence the vascularization capacities of scaffolds seeded by ADSCs. Therefore, synergistic angiogenesis promotion effects were reported by the combination of ADSCs and extracellular matrix scaffolds.

Conducting investigations demonstrate that ADSs can positively contribute to the repair of normal and pathological cutaneous wounds through the secretion of soluble factors, facilitating angiogenesis, and differentiation into different cell lines. Nonetheless, a conclusive statement about the clinical benefits of ASCs is not yet possible due to insufficient high-quality clinical investigations^56^.

Our study proved that using stem cells with or without ADM cannot enhance the process of wound healing or improve pathology indices. Injection of stem cells to wounds, unlike most other studies, could not advance wound healing. Moreover, ADM probably is not a proper carrier for transferring stem cells to the surface of the wound; however, more examinations are required in this matter. 

Most research works focusing on the application of ASC-seeded ADM scaffolds reported the positive synergistic effect of ASCs and ADM on wound healing without obvious complications. However, all the studies suffer from the lack of randomized studies with high quality and a sufficient number of patients. 

## CONCLUSION

Using ADM could enhance wound healing but using stem cells with or without ADM cannot enhance the process of wound healing or improve pathology indices. ADM probably is not a proper carrier for transferring stem cells to the surface of the wound; however, we need more examination in this matter.
